# The New Stealth Drug on the Street: A Narrative Review of Xylazine as a Street Drug

**DOI:** 10.7759/cureus.40983

**Published:** 2023-06-26

**Authors:** Joseph Pergolizzi Jr, Jo Ann K LeQuang, Peter Magnusson, Thomas L Miller, Frank Breve, Giustino Varrassi

**Affiliations:** 1 Pain Medicine, Nema Research, Inc., Naples, USA; 2 Healthcare Policy, Nema Research, Inc., Naples, USA; 3 School of Medical Sciences, Örebro University, Örebro, SWE; 4 Clinical Development, Enalare Therapeutics, Inc., Princeton, USA; 5 Pharmacy, Temple University, Philadelphia, USA; 6 Pain Medicine, Paolo Procacci Foundation, Rome, ITA

**Keywords:** "tranq", sedatives, fentanyl, overdose, street drugs, polysubstance abuse, xylazine, alpha-adrenergic receptor

## Abstract

Xylazine is an alpha-adrenergic receptor agonist approved for use only in animals with a prescription from a veterinarian. It is a powerful sedative that is slowly infiltrating the recreational street drug scene and is often used by polysubstance abusers. Known as “tranq,” it can be fatal, and xylazine-induced toxicity cannot be reversed with naloxone or nalmefene. Due to its vasoconstrictive effects, chronic use of xylazine is associated with necrotic skin lesions and general deterioration of health. Since xylazine is not approved for human use and is not scheduled as a controlled substance, there are no human studies to provide evidence of drug-drug interactions, lethal doses, or reversal protocols. Xylazine is available online without a prescription. Street drug users may take xylazine knowingly or unknowingly, as it is often combined with other illicit substances such as fentanyl. There are no rapid tests for xylazine, although there are specialty tests that can be ordered. Xylazine represents a major threat to street drug users and another challenge to emergency healthcare workers, first responders, and others who care for those who have taken this “new” street drug.

## Introduction and background

Xylazine is an α-adrenergic receptor agonist approved by the Food and Drug Administration (FDA) only as a veterinary medicine. Xylazine produces a sedative effect by triggering a rapid decrease in the release of norepinephrine and dopamine in the central nervous system [1,2]. In veterinary sedation, xylazine may be used alone or in combination with other anesthetic medications, such as ketamine. Xylazine has toxic effects in humans, who may also develop dependency [3].

Since xylazine is legally dispensed only by prescription from a veterinarian for use in animals, it is not scheduled by the Drug Enforcement Administration (DEA) as a controlled substance [1]. It is administered to animals either alone or together with other anesthetics, such as barbiturates or ketamine, intravenously, intramuscularly, or orally for sedation and relaxation. Since it was never approved for human use in any setting, there are no studies on its pharmacology in humans. Routine toxicology screens do not identify xylazine, and since xylazine is rapidly eliminated from the body, it may not be recognized as an adulterant in other illicit drugs or as part of a polysubstance cocktail [1]. Specialty labs can test for xylazine, but there are currently no rapid test options for xylazine. Case studies show that xylazine is associated with substantial morbidity and mortality, and there are no protocols for treating xylazine overdose or xylazine withdrawal. Xylazine cannot be reversed with naloxone or nalmefene [4-6]. The lethal dose for humans is not known, and there are reports of xylazine-associated deaths with only “trace amounts” of xylazine [1].

Xylazine, which is widely available online, is sneaking slowly into the supply of illicit drugs, sometimes mixed with fentanyl, heroin, or cocaine [2]. Drug overdose mortality statistics from the U.S. Census revealed that xylazine was found in 0.36% of such deaths in 2015 but had risen to 6.7% in 2020 [5]. This figure is likely understated, as xylazine is not a drug for which authorities routinely test.

An important but rarely discussed aspect of xylazine is that it is listed by the DEA as a prominent agent in drug-facilitated crimes, such as date rape, other forms of sexual assault, and robbery [7]. Since the drug was never studied in humans, there is little reliable evidence to guide first responders and emergency healthcare workers. On November 8, 2022, the FDA issued an alert urging healthcare professionals to be aware of the potential inclusion of xylazine in recreational street drugs, particularly in polysubstance abuse and mainly associated with fentanyl [8].

The purpose of this short narrative review is to summarize what is known about xylazine as a street drug and appropriate clinical considerations.

Methods

The PubMed database was searched in March 2023 for the keyword “xylazine” and results limited to papers about human use, which yielded 199 items. The authors then examined this literature and included articles about xylazine as a street drug. Google Scholar and gray literature, such as websites of the FDA and DEA, were also searched. Since xylazine as a street drug is a newsworthy topic, our Google Scholar search also yielded newspaper articles. The authors also reviewed the bibliographic references of selected articles. This is a new and evolving topic, and 21 relevant articles were found from these sources.

## Review

It would be misleading to think of xylazine as a novel drug; even human toxicity from this drug has been reported sporadically for the past decades. From 2000 to 2014, Texas Poison Centers reported a total of 76 cases of xylazine toxicity in humans, of which 64% were unintentional [9]. Nor is xylazine use confined to the United States; a systematic review of xylazine misuse found 98 cases of xylazine overdose in nine nations [3]. The most frequently reported symptoms of xylazine overdose were drowsiness, lethargy, bradycardia, hypotension, slurred speech, and, less frequently, hypertension [9]. Xylazine causes central nervous system depression and has been associated with potentially life-threatening respiratory depression [10]. Xylazine is often used recreationally as a component in polysubstance use, but there is no human drug interaction research to provide empirical knowledge as to how xylazine interacts with other drugs in humans.

Xylazine in the illicit drug stream

Xylazine is increasingly detected as an adulterant in various street drugs [11], but little is known about how the drug is infiltrating the street drug market or why. Xylazine is produced as a liquid but is sometimes available as a powder on the street; while recreational users may inhale it or take it orally, the preferred route of administration is intravenous (IV) injection [12]. While xylazine may be taken alone, it is commonly injected as part of a polysubstance cocktail, and users are not always aware of its inclusion. A street drug user in Philadelphia reported that the main appeal of xylazine, which he took knowingly, was that it extended the normally brief but intense psychoactive effects of fentanyl, making fentanyl plus xylazine (called “tranq” in Philadelphia) a popular drug combination [13]. A recreational drug user in Puerto Rico reported he first encountered xylazine (called "anestesia" in Puerto Rico) when he found his packet of heroin contained a small paper packet of a dark powder offered as a gift. The man stated that for a long time, xylazine and heroin were offered together but in separate packets so the user could control how much, if any, xylazine he wanted, but gradually the heroin was only available premixed with xylazine in proportions of about 80% xylazine to about 20% heroin [14]. Other dealers were packaging or including xylazine with cocaine in a drug combination known as a “speedball” [14].

Puerto Rico has been ground zero for xylazine, with widespread use of the tranquilizer on the streets long before it arrived in the continental United States. A study of discarded syringes in Puerto Rico more than a decade ago found that 37.6% of all syringes contained some amount of xylazine, and 90.6% of speedball syringes had xylazine [15]. A follow-up survey of street drug users in the area where syringes were collected found that only 50% of injection drug users reported that they took xylazine [15].

Xylazine is now widely detected in street drug supplies in North America. In Philadelphia, xylazine's presence in overdose deaths from heroin and/or fentanyl rose from fewer than 2% of cases in 2014 to 31% of cases in 2019 [16]. A study based on data from a drug detection service in Toronto found that xylazine was first detected in September 2020 in the Toronto area, but in 2021, 7.2% of all samples containing fentanyl and 12.5% of all samples containing methamphetamine also contained xylazine [17].

While data on xylazine are limited because the Centers for Disease Control and Prevention (CDC) do not track xylazine data [2], patterns of xylazine use appear to have an uneven geographical distribution. The highest prevalence of overdose deaths associated with xylazine in the United States occurs in Philadelphia (25.8% of all drug-related mortality), Maryland (19.3%), and Connecticut (10.2%) [13]. In 2022, a testing lab reported that it had identified xylazine in samples from 30 states [2].

Xylazine morbidity

Morbidity with chronic xylazine use has been briefly discussed in the literature, mainly emphasizing an overall physical deterioration and potentially severe cutaneous lesions [10,18]. A survey of 89 street drug users in Puerto Rico found that more than a third reported some type of skin lesion [10]. These lesions can rapidly become infected and lead to necrosis and amputation [8].

The literature contains two illustrative case reports. A 32-year-old man with a history of IV use of illicit drugs presented with nonhealing ulcers on both lower legs. He had developed a purulent wound on his left ankle approximately nine months earlier, which had spread to necrotizing lesions on both lower legs [19]. He knowingly combined xylazine with fentanyl to extend it psychoactive effects and said if he missed a vein during injection, the injection site would soon ulcerate. He was treated with antimicrobial therapy and referred to treatment for his substance use disorder [19]. In another case report, a 37-year-old woman presented with bilateral ulcers of the lower extremities, abscesses, and tibial osteomyelitis [20]. She was an IV drug user with a preference for fentanyl combined with xylazine. Her wounds necessitated five separate hospitalizations over the next eight weeks [20].

The mechanism by which xylazine causes necrotic lesions is thought to be local vasoconstriction, particularly in the lower extremities, which results in diminished perfusion to the regional skin [20]. Prolonged periods of poor perfusion combined with poor healing due to chronic substance use can lead to infections. Furthermore, these conditions occur in street drug users, many of whom experience homelessness, chaotic lifestyles, and limited access to medical care, which worsens their prognoses.

Xylazine overdose

In 98.4% of all overdose deaths associated with xylazine, fentanyl was also present [13]. In cases of polysubstance overdose, it is not always possible to determine which drug was the causative agent of death and which drugs were contributing factors. Among those overdose deaths associated with xylazine, 45.4% also involved cocaine, 28.4% benzodiazepines, 23.3% heroin, and 19.7% alcohol [13]. Xylazine toxicity in humans has been identified at doses ranging from 40 mg to 2,400 mg, and fatalities have been associated with plasma concentrations ≤ 16 mg/L, allowing for no safety margin in xylazine exposure [10].

Much of what is known about xylazine overdose comes in the form of case reports. A 27-year-old man living in a rural community purposely administered to himself a 1.5 g IV injection of xylazine and became comatose with profound hypotension and bradycardia [21]. He was taken to the hospital where he was intubated and mechanically ventilated. He made a full recovery after about 30 hours. The serum half-life of xylazine in this patient was estimated to be 4.9 hours [21]. 

In a similar case, a 19-year-old veterinary nurse accidentally injected himself with 2 g of xylazine and became comatose, hypotensive, bradycardic, and acidotic. He received immediate supportive care and made a full recovery after several hours [22]. 

A 36-year-old veterinarian injected xylazine and ketamine and developed hypertension, tachycardia, and gastrointestinal symptoms. He recovered after emergency care [23]. A 38-year-old man presented at the emergency department after accidentally irrigating both eyes with about 8 mL of xylazine (100 mg/mL) [24]. He was asymptomatic upon arrival, and both eyes were irrigated in the emergency room with isotonic crystalloid. The patient remained under observation for about two hours after exposure to xylazine, where he developed sinus bradycardia (40 to 50 beats per minute), hypotension (90/60 mmHg), and diminished consciousness. He remained in the hospital for a day and received IV fluids. His symptoms persisted for about 24 hours and then resolved; he was discharged without further adverse effects [24].

In some cases, xylazine was taken knowingly by the patient as a recreational drug. A 19-year-old man who regularly took antacids to manage epigastric symptoms presented at the emergency room with syncope and bradycardia, which could not be explained. A urine assay found xylazine, phenobarbital, ketamine, and norketamine in his system, whereupon the patient acknowledged polysubstance drug use for the past six months. He was effectively treated with IV fluids and discharged from the hospital after four days [25].

While the evidence in the literature is limited, there may be some cases in which clinicians suspect an overdose involving or solely caused by xylazine (Table 1). It is important to note that rapid drug screens do not reveal xylazine use.

**Table 1:** Cases when clinicians may suspect xylazine is causing or contributing to an overdose.

Clinical manifestation	Description	Clinical considerations
Signs similar to opioid overdose	Miosis, apnea, respiratory depression, bradycardia, hypotension	Xylazine does not respond to naloxone, but naloxone may reverse opioids if they are used in a polysubstance cocktail
Hypothermia	Chills, feeling cool, trembling	This is associated with xylazine and not cocaine or opioids
Coma, long periods of being unresponsive	Xylazine can cause users to “sleep” and become unresponsive for hours	Eight- to 72-hour periods of being unresponsive are possible
Cutaneous lesions, up to and including large necrotizing lesions, typically on lower legs	Nonhealing purulent wounds, typically with foul-smelling discharge	Cutaneous lesions occur in about one-third of chronic xylazine users and may progress rapidly

There is no evidence for guiding clinicians in xylazine overdose rescue [18]. Xylazine toxicity, which is not always known to the patient or clinician, can be treated with supportive care, such as IV fluid resuscitation, blood pressure support, atropine, and in-clinic observation because of the potential for cardiovascular adverse events [18,25]. Alpha-adrenergic antagonists, such as atipamezole or yohimbine, have been shown to effectively reverse the sedative effects of xylazine, such as poor tissue perfusion, bradycardia, or hypotension. Yohimbine is derived from an evergreen plant native to Africa and works as an α-adrenergic receptor antagonist [26]. These agents are not approved for xylazine reversal in humans, and care should be taken because high doses of yohimbine can be fatal [27]. In animals, tolazine is the antidote for xylazine and is used at the end of surgical procedures [28].

Xylazine withdrawal and detoxification

Little is known about xylazine withdrawal symptoms or how to help patients navigate these effects. A case study reports a 29-year-old woman who was hospitalized for leg ulcers due to xylazine injections; this patient had both opioid and xylazine use disorders [29]. In the hospital, she experienced what was termed “xylazine withdrawal,” which was managed with dexmedetomidine infusions. She was transitioned to phenobarbital and tizanidine and then rotated to clonidine. Her xylazine-related symptoms resolved in about four days. Meanwhile, she was transitioned to buprenorphine to manage her opioid use disorder. She was discharged from the hospital after a stay of 19 days, taking buprenorphine, clonidine, and gabapentin [29].

Another case study reported on chronic subcutaneous xylazine use for two years by a 35-year-old veterinarian who stated he experienced no cravings or withdrawal symptoms when he periodically discontinued using the drug [30]. His family contradicted this self-report and said his drug use was escalating and he was frequently found unconscious at home. When he had to give up his veterinarian practice, he was involuntarily committed, and it was found in addition to xylazine, his daily drug regimen included haloperidol 3 mg/day, lorazepam 1 mg/day, fluvoxamine 250 mg/day, biperiden 6 mg/day, and diazepam 5 mg, as needed. The detoxification protocol used by the clinic was 0.025 mg/twice daily of clonidine with close clinical monitoring, especially for blood pressure, pulse, and cardiac activity. His withdrawal symptoms overall were mild and included muscle twitching, restlessness, and fatigue. As the detoxification was ending, he also reported an increased appetite. Despite his advanced education, he exhibited serious deficits in cognitive testing after detoxification. He scored low on intelligence tests, had a poor memory, had little ability to acquire new information (although he remembered previously learned information), and had limited executive function. Social cognition scores were equivalent to those of a 14-year-old. Brain scans showed distinct cortical atrophy, but it is not clear if the cause of the brain damage was chronic xylazine use, early-onset frontotemporal dementia, or some other new-onset mental health condition [30].

Clinical considerations

Evidence-based guidance for xylazine toxicity is an urgent and unmet medical need. Many clinicians and first responders know little about xylazine, and many poison centers do not routinely test for it. Clinicians encountering xylazine in their patients have few resources. Not all veterinarians or others who come in contact with xylazine as part of their medical practice are aware of its "alter ego" as a street drug.

Because xylazine is a veterinary drug, there is a paucity of information about the use or pharmacokinetics of xylazine in humans, its potential drug-drug interactions, or xylazine rescue in humans. Xylazine can cause respiratory depression that cannot be reversed by naloxone [4-6]. Thus, when xylazine is involved in a polysubstance-induced respiratory depression, naloxone may no longer be a plausible rescue agent. Clinicians have no established “toolkit” for managing xylazine morbidity and mortality, and addiction specialists are also limited in terms of their knowledge about potential xylazine dependence. Since xylazine is overwhelming the street drug scene with no signs of abating, this has created a series of urgent and unmet medical needs (Table 2).

**Table 2:** The urgent but unmet clinical needs for treating xylazine morbidity and mortality. CDC: Centers for Disease Control and Prevention; DEA: Drug Enforcement Administration.

Need	Utility	Comments
Rapid test capacity for xylazine and broad deployment	Allow first responders, emergency professionals, and hospitals the ability to rapidly ascertain the possible presence of xylazine in overdose patients	Can be built into existing rapid drug testing kits
Track xylazine and publish data	Need to know where the drug is most likely to be found, patterns of use, and how it is being diverted	CDC currently does not track this information
Detoxification protocols	Guidance for clinicians to help patients navigate acute and longer-term withdrawal from xylazine	Requires more data, expert consensus
Identification of diversion pathways	To help stem the tide of xylazine it is important to know where it is coming from—diversion from pharmaceutical companies? Clandestine labs? Veterinary clinics? Smuggling?	Xylazine has crept into the recreational drug supply
Schedule xylazine as a controlled substance	The DEA must schedule xylazine as a controlled substance	Right now, xylazine has a murky legal status
Get xylazine off the internet	No longer permit xylazine sales online (see above)	Requires veterinary prescription but some sites may waive this
Alert street users about the dangers of xylazine	Many street users are not aware of xylazine or its risks	Emphasize necrotizing skin lesions, ideally with images
Develop protocols for xylazine morbidity	Protocols are needed for treating respiratory depression as well as skin ulcers	Requires more data, expert consensus
Study the pharmacology of xylazine in humans	Needed to better understand potentially dangerous dose ranges and pharmacological parameters	This information is needed to help guide treatment
Study the interaction of xylazine with other drugs, particularly street drugs	It appears now that xylazine is primarily taken as an adjuvant in polysubstance use cocktails but we do not know if these other drugs potentiate xylazine	Needed to treat polysubstance overdose patients
Greater awareness about xylazine among healthcare professionals	May aid in appropriate diagnosis and speed up time-to-treatment	Facilitate diagnosis and treatment
Determine rescue protocol/agent(s)	Naloxone and nalmefene do not reverse xylazine overdose	A rescue agent could help save lives

Discussion

The stealthy intrusion of xylazine into the illicit drug market underscores three significant shortfalls in reducing harms due to illicit drugs. First of all, the fact that xylazine is not a controlled substance confounds law enforcement efforts. Second, xylazine is not well understood by clinicians and has never been studied in humans. And finally, healthcare professionals are often unfamiliar with this agent, and we have no protocols or even consensus guidelines as to managing xylazine toxicity.

A profile of the “typical” xylazine user does not exist, but from scant data, we can cobble together a sketch that such users tend to be young men who use illicit IV drugs and that xylazine is most commonly part of a polysubstance combination either with fentanyl or cocaine (“speedball”), although other combinations have been reported as well, such as with methamphetamine [12]. It is not clear if street drug users in general are aware of taking xylazine. It is also not known whether there is an economic or other motive for cutting it into illicit fentanyl and heroin.

It is unclear from where xylazine is being sourced for street traffic. Individual users may purchase xylazine online, but larger quantities may be difficult to access. Pharmaceutical xylazine is available in liquid form, and it takes considerable skill to mix liquid xylazine into heroin powder in such a way that both agents remain pharmacologically intact [14]. It is suspected that mixing xylazine into other drugs is work done by local dealers [14].

Xylazine is the latest, but not likely the last, significant challenge in harm reduction for street drug users. Since sporadic reporting of xylazine abuse and toxicity has been known for over a decade, our current state is due in part to a lagging and lackluster response as much as an insidious new drug entering the drug supply. It is imperative to respond more quickly and effectively to these stealth drugs creeping into illicit drug supplies.

## Conclusions

Few clinicians are aware of xylazine toxicity in street drug users, and no healthcare professional or first responder has adequate tools to treat it. Xylazine is a veterinary tranquilizer that is increasingly detected in the illicit drug stream, typically mixed with fentanyl, where it allegedly prolongs the psychoactive effects, or cocaine. Xylazine is not a controlled substance and was never evaluated in humans, so very little is known about its pharmacological effects or drug-drug interactions. The CDC does not track xylazine statistics, although it has been detected in drug samples from 30 states, and its role in fatal overdoses, whether causal or contributory, is increasing. There are urgent unmet medical needs related to xylazine, particularly in protocols for how to manage xylazine overdose, rescue, detoxification, and awareness.
